# Accelerometer Thresholds for Estimating Physical Activity Intensity Levels in Infants: A Preliminary Study

**DOI:** 10.3390/s24144436

**Published:** 2024-07-09

**Authors:** Mustafa A. Ghazi, Judy Zhou, Kathryn L. Havens, Beth A. Smith

**Affiliations:** 1 Division of Developmental-Behavioral Pediatrics, Children’s Hospital Los Angeles, Los Angeles, CA 90027, USA; mghazi@chla.usc.edu; 2 Division of Biokinesiology and Physical Therapy, Ostrow School of Dentistry, University of Southern California, Los Angeles, CA 90089, USA; 3 Developmental Neuroscience and Neurogenetics Program, The Saban Research Institute, Children’s Hospital Los Angeles, Los Angeles, CA 90027, USA; 4 Department of Pediatrics, Keck School of Medicine, University of Southern California, Los Angeles, CA 90033, USA

**Keywords:** physical activity intensity, wearable accelerometer, infant, threshold, validation

## Abstract

Lack of physical activity (PA) at a young age can result in health issues. Thus, monitoring PA is important. Wearable accelerometers are the preferred tool to monitor PA in children. Validated thresholds are used to classify activity intensity levels, e.g., sedentary, light, and moderate-to-vigorous, in ambulatory children. No previous work has developed accelerometer thresholds for infancy (pre-ambulatory children). Therefore, this work aims to develop accelerometer thresholds for PA intensity levels in pre-ambulatory infants. Infants (n = 10) were placed in a supine position and allowed free movement. Their movements were synchronously captured using video cameras and accelerometers worn on each ankle. The video data were labeled by activity intensity level (sedentary, light, and moderate-to-vigorous) in two-second epochs using observational rating (gold standard). Accelerometer thresholds were developed for acceleration and jerk using two optimization approaches. Four sets of thresholds were developed for dual (two ankles) and for single-worn (one ankle) accelerometers. Of these, for a typical use case, we recommend using acceleration-based thresholds of 1.00 m/s to distinguish sedentary and light activity and 2.60 m/s to distinguish light and moderate-to-vigorous activity. Acceleration and jerk are both suitable for measuring PA.

## 1. Introduction

Engaging in physical activity plays a significant role in improving and maintaining health. Higher levels of physical activity at an early age (0–4 years) are associated with improvements in adiposity, bone and skeletal health, psycho-social health, cardio-metabolic health, cognitive development, and motor skills development [1]. Measuring physical activity and its relationship to adiposity is one of the concerns, owing to the rise in obesity rates, in both adults and children. Increased weight gain during infancy has been associated with a higher risk for obesity later in life [2]. While there is interest in measuring physical activity in infancy, the field is currently lacking a validated measure of amount and intensity of physical activity in pre-ambulatory infants.

Physical activity intensity can be measured through one of many direct or indirect standardized measures, namely, doubly labeled water (DLW), maximal oxygen consumption (VO_2MAX_), heart rate (HR) monitoring, observational ratings (video coding), wearable accelerometers, and self-reported diaries (see Table 1). The advantages and limitations of each method are listed in detail in Table 1, but we will briefly mention observational ratings and wearable accelerometers here. Observational ratings are not practical for widespread use, and no such scales exist for infants (see Table 1). Wearable accelerometers have become a widely used method for physical activity intensity classification over the last two decades, e.g., Sirard et al. [3]. Their relatively modest cost [4] and convenience of use make them a popular tool, despite some limitations (see Table 1). Accelerometer thresholds for activity intensity levels have been developed for multiple populations of children, e.g., by Sirard et al. and Trost et al. [3,5] but not yet for pre-ambulatory infants.

The measurement of physical activity intensity using wearable accelerometers is a two-step process. First, accelerometer data are integrated or summed (often called activity counts) over intervals of time, also known as epochs. Each epoch is then classified based on its numerical value by using pre-defined threshold levels for different types of physical activity, most notable being sedentary, light, moderate, and vigorous activity. These thresholds are developed and validated for various population groups and activity types using one or more established physical activity intensity measurement methods, such as VO_2MAX_, HR monitoring, or observational rating, as a gold-standard comparison. An example of this process is described in Evenson et al. [13]. It is important to note that thresholds developed for one population or activity are not applicable to another without validation.

Wearable accelerometers have been used to study infants, ambulatory toddlers, and older children. Accelerometer thresholds for physical activity have been developed for ambulatory toddlers and older children [5,10]. However, while infants, whose movements are different, have been a focus of research, accelerometer thresholds for their physical activity have not been developed. A 2022 systematic review identified 76 research articles that used wearable sensors for studying physical activity of children and adolescents [14]. Of these, only two studied an infant population, but both focused solely on detecting sleep versus wake time [15,16]. A 2020 systematic review identified 19 research articles that classified infant and toddler physical activity using accelerometers [17], out of which only five articles studied infants. Of these five articles, one used thresholds developed for 3–5 year olds [18], one computed physical activity intensity levels for mothers but not infants [19], one computed minimum accelerometer wear time [2], one studied sleep versus active time [20], and one studied sleep/wake and infant/mother activity patterns [21]. Another systematic review identified 62 research articles that classified children’s physical activity using accelerometers [22]. Out of these, thirteen studied infants, but eight of these only studied sleep versus wake states [15,23,24,25,26,27,28,29]. Of the remaining five articles, two detected posture [30,31], two classified activity by type [32,33], and one quantified characteristics of leg movements [34]. Even the most recent articles focus on sleep/wake states, e.g., Liu and Benjamin-Neelon [35] and Bucko et al. [36].

Overall, we are aware of four examples of work that could be considered close to developing physical activity intensity thresholds for infants. Ricardo et al. [37] used average daily acceleration as an estimate of the total volume of movement or physical activity; however, the authors did not validate that higher daily acceleration values corresponded to a higher total volume of infant movement or physical activity. Bucko et al. [36] used a similar approach by using average acceleration from 7 AM to 7 PM to quantify daily physical activity, also without any validation. Thelagathoti et al. [38] used acceleration magnitude to study similarities in physical activity patterns between infants but without validating against an actual measure of physical activity. Thureen et al. [39] validated a custom force plate-based analysis to estimate the contribution of physical activity to total energy expenditure in infants; however, their work does not include accelerometer measurement.

Measuring the amount and intensity of physical activity produced by infants is important for assessing health status. There is a lack of validated wearable accelerometer-based methods to quantify the physical activity intensity of pre-ambulatory infants. In this context, the goal of this study is to derive accelerometer thresholds for wearable sensor classification of infant physical activity intensity by using observational rating of physical activity intensity as a gold standard. The intensity levels are sedentary, light, and moderate-to-vigorous (MV). The novel contribution of this preliminary study paper is the development and recommendation of variables and thresholds for wearable sensor classification of infant physical activity intensity.

## 2. Materials and Methods

### 2.1. Population and Setting

These data were part of a larger study [40,41] about the day-to-day variability of full-day wearable sensor recordings across 7 days in infants who were typically developing (TD group) and infants who were at risk for neurodevelopmental disabilities (AR group). In the larger study, approximately 5 min of videos of the infants was recorded on their first day, and sensor data were recorded over 7 days. For this paper, we analyzed data from a subset of 10 infants. These were all the infants (*n_TD_* = 6, *n_AR_* = 4) for whom both the video data and wearable sensor data were available (5.73 min mean), such that they were overlapping and were synchronized in time. The infants’ ages ranged from 31 to 433 days (adjusted for prematurity in the AR group). The details of the infants are presented in Table 2. The data were collected in the infants’ homes. During the data collection session, the infants were placed in supine to start. If they rolled out of supine, they were repositioned back into supine by the researcher. Data were recorded while the infants were awake and alert. The caregivers were allowed to interact with their infants but were requested to refrain from physically touching them.

**Figure 1:**
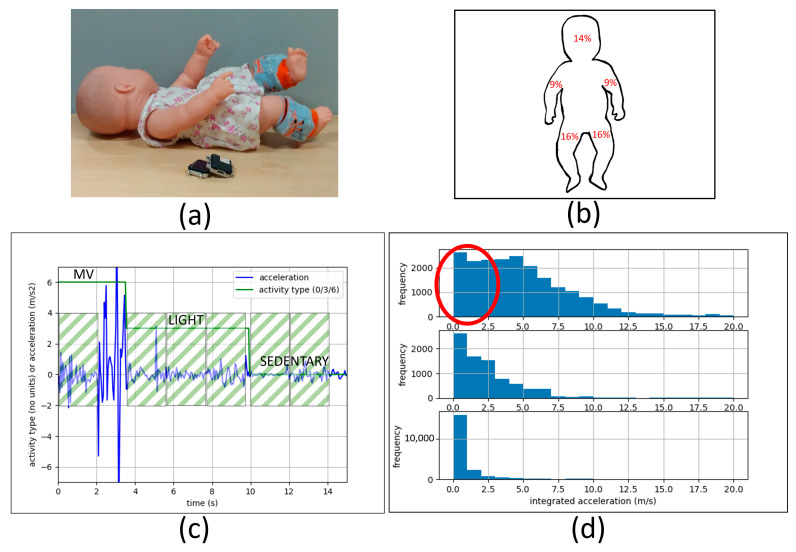
Data collection and processing. (**a**) Illustration of sensor attachment on the infants’ ankles along with two more next to the body; snug-fitting leg warmers with pockets were used to hold the sensors (sensor model: Opal Version 1). (**b**) Infant body segment contributions as used for observational rating (video coding). (**c**) Illustration of valid (green, diagonal stripes) 2-s non-overlapping windows used for computation; the valid windows always start at the start of an activity region and are completely within a single activity intensity region; areas at the end of each activity region that were less than the 2-s window length were not used for computation. (**d**) Histograms for sedentary, light, and moderate-to-vigorous activity counts (all participants); the *x*-axis is a derived quantity (integrated acceleration); histogram bin size is 1 m/s; the circled region shows a potential area where the data belong to moderate-to-vigorous activity but would appear to be light activity (according to the accelerometers).

For each infant, anthropometric data were collected (weight, body length, head circumference, thigh length, shank length, thigh circumference, and shank circumference) in addition to the video and sensor data. The videos were recorded at 30 Hz using a video camera placed above and at the head or feet of the infant so that the infant’s whole body was visible. Wearable inertial sensors were used to collect actively synchronized tri-axial accelerometer, gyroscope, and magnetometer data at 20 Hz (Opal sensor, version 1, APDM Inc., Portland, OR, USA). The sensor data were stored in the onboard memory within the sensor modules themselves. Two sensors were used, one at each ankle of the infant (see Figure 1a). The sensors were attached to the ankles using custom leg warmers in various sizes that had pockets for the sensors. After the start of the video recording and in view of the camera, the researcher pushed a button on one of the sensors to create a time stamp in the sensor data file. This allowed for the alignment of the wearable sensor and video data post-collection. The data session length, after the sensor and video data were aligned, ranged from 2.71 to 8.45 min (see Table 2).

### 2.2. Observational Rating (Video Coding)

Observational rating was performed by trained coders who observed and coded the synchronized video section using ELAN software version 5.8-6.2 (The Language Archive, Nijmegen, The Netherlands). Before coding, the coders had to obtain greater than 80% reliability with training files. Twenty percent of the data was coded by two coders to assess inter-rater reliability and ensure the reliability remained above 80%. Any discrepancies were resolved by discussion, bringing in a third rater if needed.

The videos were assessed in epochs that were at least 2 s long (more details below). Each epoch was coded for one of three activity intensity levels, sedentary (SED), light, or moderate-to-vigorous (MV), similar to those defined by Trost et al. (2012) [5]. Sedentary activity level was defined as having a static posture and less than 15 percent total segment movement. Light activity level was defined as having a static or changing posture and 15 to 30 percent total segment movement. Moderate-to-vigorous activity was defined as having static or changing posture with more than 30 percent total segment movement. Each limb and the head were considered as segments, as shown in Figure 1b. These defined percentage contributions of each segment were based on standardized estimates of body surface area for infants under 12 months of age. Whether the posture was static or changing was determined by whether or not the trunk was moving. For example, if one arm was moving and nothing else, the classification would be sedentary. If, however, one arm and one leg or one arm and the head were moving, then the classification would be light. The thresholds for activity levels (below 15%, 15–30%, above 30%) were selected such that, for light activity, either one leg or two of the other segments (arm or head) should be moving, and for MV activity, both legs or any three segments (leg, arm, head) should be moving. For pre-ambulatory infants, the majority of their physical activity is produced using limb movements. When they are in supine or sitting, their limbs are often moving, while their trunk and head are often still. When they are crawling or rolling, their limbs, trunk, and head are all moving. Consequently, sensors placed on the limbs should adequately capture the physical activity of pre-ambulatory infants.

For video coding, each epoch was at least 2 s long. The start of a new epoch was noted if the infant moved to a new activity intensity level and maintained that for at least 2 s. The end of the epoch was defined by the start of a new epoch as described above.

### 2.3. Wearable Accelerometer Data Processing

Derived quantities were computed from the raw acceleration data from the wearable accelerometers. A derived quantity for a given epoch can be compared with the thresholds to determine the activity intensity level or, initially, compared with the corresponding observational rating score to determine the thresholds. Two derived quantities were computed: area under the acceleration–time curve and area under the jerk–time curve. The former is analogous to the classic accelerometer counts and represents the total change in velocity. The latter is analogous to the total change in acceleration (and force). Area under the acceleration–time curve is similar to activity “counts” from the most widely used sensors for measuring the intensity of physical activity, i.e., ActiGraph sensors [42], and “counts” from these sensors have been well-established in quantifying physical activity. Jerk is the time-derivative of acceleration, and time derivatives can be better at capturing changes in a quantity. We hypothesized that the area under the jerk–time curve could perform better than the area under the acceleration–time curve because jerk is representative of the change in force generated by the limb over time.

Activity “counts” from ActiGraph algorithms use raw analog-to-digital (ADC) values of 0–128, equivalent to 0–20.9 m/s^2^ acceleration based on the first ActiGraph model AM7164 [42,43]. Data from another accelerometer with a different ADC and with a different resolution and range will have to be converted to this scale for comparison. In contrast, our approach captures the same aspects of movement but with universal units of acceleration that are independent of ADC resolution and range. Activity “counts” from ActiGraph algorithms are computed by summing the magnitudes of the acceleration values in a given epoch of time with a fixed sensor sampling rate of 10 Hz [42]. This is identical to the area under the acceleration–time curve. In contrast, our approach computes the actual area under the acceleration–time curve, independent of the sensor sampling rate. Thus, our approach is more universally applicable to various sensor models and manufacturers, regardless of the ADC resolution and range or the sampling rate.

As a first step, the magnitude *a_M_* of the three-dimensional acceleration vector [*a_x_ a_y_ a_z_*] was computed at each instant *i*:

(1) $$a_{M,i}=\sqrt{{\left(a_{x,i}\right)}^{2}+{\left(a_{y,i}\right)}^{2}+{\left(a_{z,i}\right)}^{2}}$$

where *a_x,i_*, *a_y,i_*, and *a_z,i_* are the three components of the tri-axial accelerometer output at instant *i*.

In the second step, the gravity component was removed from the acceleration magnitude *a_M,i_* to obtain a gravity-independent magnitude of acceleration *a_IND,i_* at each instant *i*:

(2) $$a_{IND,i}=\;a_{M,i}-a_{G}$$

The baseline acceleration due to gravity *a_G_* was computed by taking the mean acceleration from “static” segments of the data (Figure 1). Static segments were defined as continuous regions of 10 data points (0.5 s duration) where the difference between the minimum and maximum of *a_M_* was less than or equal to 0.1 m/s^2^ (10 mg). This threshold was determined after a visual observation of the data. This is comparable to the 0.128 m/s^2^ (13 mg) threshold used by the UK Biobank study on wearable accelerometers [44]. If no static regions were detected, then *a_G_* was taken to be the median of the data, as in our previous work in wearable accelerometers [34].

For the third step, the gravity-independent magnitude of acceleration *a_IND,i_* was scaled based on infant leg length (thigh length + shank length). This is because longer legs would result in a higher acceleration for the same angular movement. Therefore, adjusted acceleration *a_ADJ,i_* was computed using the following equation:

(3) $$a_{ADJ,i}=\;\frac{a_{IND,i}}{\frac{leg\;length}{baseline\;leg\;length}}$$

where the baseline leg length was 26.5 cm for infants aged 4 months, specifically, median rump-sole length of 3–5 month-old infants [45]. The age of 4 months was selected as the baseline as, typically by this age, infants begin to demonstrate greater head control and more controlled arm and leg movements.

In the fourth step, the derived quantities *c_A,i_* (area under the acceleration–time curve) and *c_J,i_*, (area under the jerk–time curve) were computed by numerical integration:

(4) $$c_{A,i}=\int_{t=t_{i}-2}^{t_{i}}a_{ADJ,i}\;dt\approx \sum_{t=t_{i}-2}^{t_{i}}a_{ADJ,i}∆t$$

(5) $$c_{J,i}=\int_{t=t_{i}-2}^{t_{i}}j_{i}\;dt\;\approx \;\sum_{t=t_{i}-2}^{t_{i}}j_{i}∆t$$

(6) $$j_{i}=\;\frac{a_{ADJ,i}-a_{ADJ,i-1}}{∆t}$$

where *Δt* is the time elapsed between two instances (inverse of sensor sampling rate), and *j_i_* is the instantaneous jerk. The computation for *c_A,i_* and *c_J,i_* was performed using 2-s intervals (40 data points), using non-overlapping windows of data. Non-overlapping windows were used to ensure that each instance of *c_A,i_* and *c_J,i_* was an independent data point. This interval size was selected because this was the minimum epoch size used in the gold standard (video coding). Only windows of data that were wholly contained within one of the three activity intensity regions were used (see Figure 1c). If the end of an activity region had a region shorter than the 2-s window size, it was not included in the computation (see Figure 1c).

For the fifth and final step in processing accelerometer data, the left and right leg derived quantities were summed together, if needed, to obtain a total derived quantity *c_TOTAL,i_* at each instant *i*:

(7) $$c_{\;TOTAL,\;i}=\;c_{LEFT,\;i}+c_{RIGHT,\;i},\;or,\;c_{LEFT,\;i},\;or,\;c_{RIGHT,\;i}$$

For the development of thresholds for the sensors on both legs, left leg only, or right leg only. We predicted that using sensors on both legs would better represent the physical activity levels of the infants compared to a sensor on a single leg. However, we wanted to compare two sensors to only one sensor, as using only one sensor requires less resources.

The sensor data were aligned to observational rating (video coding) as follows. For observational rating, ELAN software version 5.8-6.2 generated a text file. This file noted the start time, end time (to the nearest millisecond), and activity label of each physical activity bout. For the sensor data, activity labels were generated to the corresponding or closest time points in the sensor data.

The sensor data were downloaded from the sensor modules using Motion Studio software version 1.0 (APDM Inc. Portland, OR, USA). The sensor and video data alignment and acceleration magnitude *a_M_* computation (Equation (1)) were performed using custom code in MATLAB (MathWorks, Natick, MA, USA). The remaining processing (baseline, derived quantities, threshold determination, etc.) was performed with custom python scripts.

### 2.4. Threshold Determination Using True Positive Rate

Activity intensity thresholds were determined based on the derived quantity *c_TOTAL,i_* (acceleration or jerk) for both legs, left leg only, and right leg only. The data for this step consisted of a collection of 2-s epochs, which were classified by activity intensity level according to the gold standard (observational rating). Each 2-s epoch was also classified by activity intensity level according to the derived quantity threshold (accelerometer). This classification of epochs is illustrated in Table 3.

This step of threshold determination was divided into two parts: a primary optimization step and a secondary optimization step. During each optimization step, an assumed threshold was incrementally adjusted until an optimum value of the relevant cost function was obtained. For each quantity (acceleration or jerk), the primary step was to determine the threshold between sedentary and active (including both light and MV) phases of movement. The threshold was determined by optimizing for the maximum true positive (TP) rate for both classes.

The maximum TP rate was obtained by minimizing the following cost function:

(8) $$cost\;function=\left|{TP}_{SED}-{TP}_{ACTIVE}\right|$$

(9) $${TP}_{SED}\;=\;\frac{{SED}_{SED}}{{SED}_{SED}+{SED}_{ACTIVE}}\times 100\;$$

(10) $${TP}_{ACTIVE}\;=\;\frac{{ACTIVE}_{ACTIVE}}{{ACTIVE}_{SED}+{ACTIVE}_{ACTIVE}}\times 100$$

This cost function works as follows. If the threshold is set too low, then *TP_SED_* would be high, and *TP_ACTIVE_* would be low. If the threshold is increased such that it is just right, then *TP_SED_* would be the same as *TP_ACTIVE_*. If the threshold is set too high, then *TP_SED_* would be low, and *TP_ACTIVE_* would be high for active periods of movement.

The secondary step was to determine the threshold between the light and MV classes of movement. In this case, we considered the MV class to be more important than the light class and tried to optimize accordingly (considering *TP_MV_* and not *TP_LIGHT_* in the cost function). Given the overlap in data, we had to make the choice between knowing with certainty the light class or MV class. We considered MV more important because, with healthy physical activity guidelines for children and adolescents, it is typically the MV class of activity that is referred to. Therefore, the cost function was set to be such that it would result in a *TP_MV_* rate that matched the TP rate of the sedentary periods of movement.

(11) $$cost\;function=\left|{TP}_{MV}-{TP}_{FIXED}\right|$$

(12) $${TP}_{MV}=\;\frac{{MV}_{MV}}{{MV}_{SED}+{MV}_{LIGHT}+{MV}_{MV}}\times 100$$

(13) $${TP}_{FIXED}=\;constant\;$$

where *TP_FIXED_* was obtained in the primary optimization step above, numerically equal to *TP_SED_* based on the optimum sedentary/active threshold. Note that *TP_FIXED_* was a fixed value and did not change during this secondary step.

### 2.5. Threshold Determination Using Predicted Activity Proportion

Similar to the section above for true positive rate, activity intensity thresholds were determined based on the derived quantity *c_TOTAL,i_* (acceleration or jerk) for the sensors on both legs, on the left leg only, and on the right leg only. For the primary step, the threshold between sedentary and active (including both light and MV) phases was computed for each derived quantity (acceleration or jerk). In this case, the threshold was determined by optimizing for the proportion of activity events predicted by the accelerometer data (including both true positives and false positives) to match the proportion of activity events detected by the gold standard (observational rating). For example, if the gold standard detected 40% sedentary and 60% active activity levels, then the threshold would be adjusted until the accelerometer data would also predict 40% sedentary and 60% active activity levels. Thus, for predicted activity proportion (PAP), we optimized the cost function:

(14) $$cost\;function=\left|∆sedentary\right|\times 100+\left|∆active\right|\times 100$$

(15) $$∆sedentary=\frac{\left({SED}_{SED}+{SED}_{ACTIVE})-{(SED}_{SED}+{ACTIVE}_{SED}\right)}{\left({SED}_{SED}+{SED}_{ACTIVE}\right)}$$

(16) $$∆active=\;\frac{\left({ACTIVE}_{SED}+{ACTIVE}_{ACTIVE})-{(SED}_{ACTIVE}+{ACTIVE}_{ACTIVE}\right)}{\left({ACTIVE}_{SED}+{ACTIVE}_{ACTIVE}\right)}$$

The cost function works as follows. The total number of activity events in each category must match the gold standard (observational rating). The count from each category was converted to a percentage so as to give equal weight to each category. The hypothesis behind this cost function was that, even though there will be some false positives, on average, the total number of classified activity events in each class (true positives and false positives) would be similar to the gold standard (observational rating).

The secondary step was to determine the threshold between the light and MV classes of movement. In this case, the same cost function as above was used but for light and MV activity classes:

(17) $$cost\;function=\left|∆light\right|\times 100+\left|∆MV\right|\times 100$$

(18) $$∆light=\frac{\left({LIGHT}_{SED}+{LIGHT}_{LIGHT}+{LIGHT}_{MV}\right)-{(SED}_{LIGHT}+{LIGHT}_{LIGHT}+{MV}_{LIGHT})}{\left({LIGHT}_{SED}+{LIGHT}_{LIGHT}+{LIGHT}_{MV}\right)}\;$$

(19) $$∆MV=\frac{\left({MV}_{SED}+{MV}_{LIGHT}+{MV}_{MV}\right)-{(SED}_{MV}+{LIGHT}_{MV}+{MV}_{MV})}{\left({MV}_{SED}+{MV}_{LIGHT}+{MV}_{MV}\right)}$$

### 2.6. Measures of Evaluation Used

Measures of evaluation were used or developed in order to gage the effectiveness of using the different derived quantities (acceleration or jerk) and optimization methods (TP rate or PAP). As discussed above, the TP rate, or sensitivity, for a particular activity class is the proportion of activity events that are correctly predicted by the accelerometer data. For example, the TP rate for the MV class is the proportion of the MV activity events that are correctly predicted by the accelerometer data:

(20) $${true\;positive\;rate}_{MV}=\;\frac{{MV}_{MV}}{{MV}_{SED}+{MV}_{LIGHT}+{MV}_{MV}}\times 100$$

The true negative (TN) rate, or specificity, for a particular class is the proportion of activity events, not belonging to that class, that are correctly predicted by the accelerometer data as not being part of that class. For example, the TN rate for the MV class is the proportion of non-MV activity events that are correctly predicted as not being part of the MV class by the accelerometer data:

(21) $${true\;negative\;rate}_{MV}=\;\frac{{SED}_{SED}+{SED}_{LIGHT}+{LIGHT}_{SED}+{LIGHT}_{LIGHT}}{{SED}_{SED}+{SED}_{LIGHT}+{SED}_{MV}+{LIGHT}_{SED}+{LIGHT}_{LIGHT}+{LIGHT}_{MV}}\times 100$$

As discussed above, the predicted activity proportion (PAP) for a particular class is the proportion of total activity events (all activity events from sedentary, light, and MV activity classes) that were predicted to be part of that class by the accelerometer data. This includes correct and incorrect predictions. For example, the PAP for the MV class is the proportion of total activity events (sedentary, light, MV) that are predicted to be part of the MV class by the accelerometer data:

(22) $${predicted\;activity\;proportion}_{MV}=\frac{{SED}_{MV}+{LIGHT}_{MV}+{MV}_{MV}}{{SED}_{SED}+{SED}_{LIGHT}+{SED}_{MV}+{LIGHT}_{SED}+{LIGHT}_{LIGHT}+{LIGHT}_{MV}+{MV}_{SED}+{MV}_{LIGHT}+{MV}_{MV}}\times 100$$

The minimum true positive rate (MTPR) is the minimum TP rate predicted by the accelerometer data when considering all the relevant activity classes. For example, for the primary optimization, the MTPR is defined as follows:

(23) $${minimum\;true\;positive\;rate}_{primary}=\min({TP}_{SED},{TP}_{ACTIVE})$$

Given that we focused on the MV activity classification over the light activity classification, as discussed previously, for the secondary optimization, the MTPR is defined as the minimum of the TP of the sedentary and MV activity classes:

(24) $${minimum\;true\;positive\;rate}_{secondary}=\min\left({TP}_{SED},{TP}_{MV}\right)\;$$

The PAP match rate (PMR) is defined as the total deviation from the gold standard (video) PAP. For example, for the primary optimization, the PMR is defined as follows:

(25) $${PAP\;match\;rate}_{primary}\;=100-\left(\left|{PAP}_{SED,VIDEO}-{PAP}_{SED,PREDICTED}\right|+\left|\;{PAP}_{ACTIVE,VIDEO}-{PAP}_{ACTIVE,PREDICTED}\right|\right)$$

Similarly, the PMR for the secondary optimization is defined as follows:

(26) $$\begin{array}{l} {PAP\;match\;rate}_{secondary}\\ \;\;\;\;\;\;\;\;=100\\ \;\;\;\;\;\;\;\;-(\left|{PAP}_{SED,VIDEO}-{PAP}_{SED,PREDICTED}\right|\\ \;\;\;\;\;\;\;\;+\left|{PAP}_{LIGHT,VIDEO}-{PAP}_{LIGHT,PREDICTED}\right|\\ \;\;\;\;\;\;\;\;+\left|{PAP}_{MV,VIDEO}-{PAP}_{MV,PREDICTED}\right|) \end{array}$$

### 2.7. Validation

After developing the thresholds by using the measures of evaluation described in the previous section, we validated the thresholds by using the same measures of evaluation. The data that we used to evaluate the thresholds were independent of the data we used for determining the thresholds. We used data from eight infants for developing the thresholds and data from the remaining two infants (one TD, one AR) for the evaluation of the thresholds.

## 3. Results

**Figure 2:**
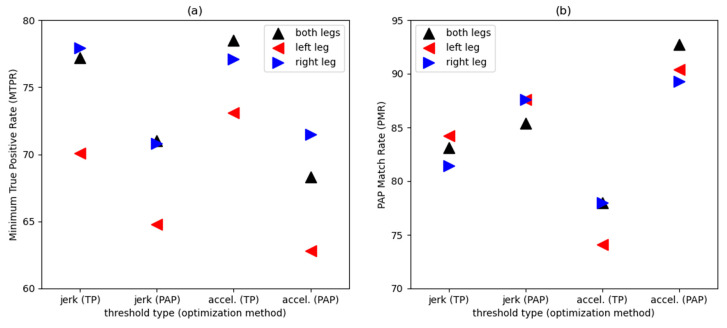
Illustrated comparison of the performance of the thresholds for the four approaches. Based on combinations of jerk–time and acceleration–time derived quantities and the true positive (TP) rate and predicted activity proportion (PAP) optimization methods (*x*-axis). Based on validation dataset to demonstrate performance of thresholds. Performance score on the *y*-axis is based on the overall rating and secondary optimization (see Table 4, Table 5 and Table 6). Higher score indicates better performance. (**a**) Measure of evaluation used is the minimum true positive rate (MTPR). As expected, in general, thresholds based on TP rate perform better. (**b**) Measure of evaluation used is the PAP match rate (PMR). As expected, in general, thresholds based on PAP match rate perform better.

Thresholds were determined for two derived quantities (jerk and acceleration). For each derived quantity, two optimization methods were used as described in the previous section (TP and PAP). The resultant thresholds for the four approaches are listed in Table 4 for both legs, Table 5 for the left leg, and Table 6 for the right leg. With the primary optimization step, the sedentary/active threshold was determined, dividing the activity intensity into two classes. In the case of both legs, this threshold was 27.0 m/s^2^ (TP) and 18.0 m/s^2^ (PAP) for jerk and 1.30 m/s (TP) and 1.00 m/s (PAP) for acceleration. With the secondary optimization step, the light/moderate-to-vigorous threshold was determined, dividing the activity intensity level into a total of three classes. This threshold was 41.0 m/s^2^ (TP) and 56.0 m/s^2^ (PAP) for jerk and 1.80 m/s (TP) and 2.60 m/s (PAP) for acceleration. An overall comparison of the performance of the thresholds is illustrated in Figure 2, with results for both legs, left leg, and right leg.

In the case of the sensor worn on the left leg only, the sedentary/active threshold was 10.0 m/s^2^ (TP) and 8.00 m/s^2^ (PAP) for jerk and 0.600 m/s (TP) and 0.500 m/s (PAP) for acceleration. The light/moderate-to-vigorous threshold was 16.0 m/s^2^ (TP) and 27.0 m/s^2^ (PAP) for jerk and 0.800 m/s (TP) and 1.30 m/s (PAP) for acceleration. In the case of the sensor worn on the right leg only, the sedentary/active threshold was 11.0 m/s^2^ (TP) and 7.00 m/s^2^ (PAP) for jerk and 0.400 m/s (TP) and 0.300 m/s (PAP) for acceleration. The light/moderate-to-vigorous threshold was 17.0 m/s^2^ (TP) and 24.0 m/s^2^ (PAP) for jerk and 0.800 m/s (TP) and 1.00 m/s (PAP) for acceleration.

The general measures of evaluation (based on the validation data) for each of the four approaches are listed in Table 7 for both legs, Table 8 for the left leg, and Table 9 for the right leg. These include the TP rate, TN rate, and predicted activity proportion as detailed in the previous section. Although each approach was only optimized for either the TP rate or the PAP, all three measures of evaluation were computed for each of the four approaches.

To determine the best approach for determining the thresholds, based on the combination of derived quantity and optimization method, the MTPR and PMR evaluation metrics were computed (based on the validation dataset). These are listed in Table 4, Table 5 and Table 6 as overall rating. Each overall rating row represents one evaluation metric for the four approaches considered. One selection was made from each row to determine the one best approach for that particular evaluation metric. The best approach is highlighted for each row. For the sensors on both legs (Table 4), thresholds based on acceleration were best in each approach, but this trend is not consistent for the sensors worn on the left leg only (Table 5) or right leg only (Table 6). In all three cases (Table 4, Table 5 and Table 6), the acceleration threshold is mostly only marginally better than the jerk threshold and vice versa. Unexpectedly, thresholds with TP optimization do not always have the highest MTPR, and thresholds with PAP optimization do not always have the highest PMR (Table 4, Table 5 and Table 6). Again, these differences between the two types of optimization are mostly only marginal. An illustration of the overall rating based on secondary optimization is shown in Figure 2.

## 4. Discussion

While activity count thresholds exist for classifying physical activity as sedentary, light, or moderate-to-vigorous using accelerometers in ambulatory toddlers, we are the first to validate accelerometer thresholds for classifying physical activity as sedentary, light, or moderate-to-vigorous using accelerometers in pre-ambulatory infants. This is a necessary distinction, as pre-ambulatory infants and ambulatory toddlers move in very different ways.

Four sets of thresholds were determined (two for jerk and two for acceleration) for each of the three sensor wearing arrangements (see Table 4, Table 5 and Table 6). In the case of the sensors worn on both legs, for the primary optimization step, the jerk PAP threshold was 33.0% lower than the jerk TP threshold. Similarly, the acceleration PAP threshold was 23.1% lower than the acceleration TP threshold. For the secondary optimization step, the jerk PAP threshold was 36.6% higher than the jerk TP threshold. Similarly, the acceleration PAP threshold was 44.4% higher than the acceleration TP threshold. These are not insignificant differences in thresholds. Similar trends were observed in the thresholds for the sensors worn on a single leg (Table 5 and Table 6). This shows that, in general, for PAP, light/sedentary threshold is always lower, and sedentary/MV threshold is always higher.

When evaluated with the independent data, we expected the thresholds based on TP optimization to perform better for the MTPR metric (Table 4, Table 5 and Table 6). We also expect the thresholds based on PAP optimization to perform better for the PMR metric. As expected, when optimized by TP, the jerk (TP) and acceleration (TP) usually presented the highest true positive rates for sedentary and moderate-to-vigorous activity (see overall rating for MTPR rows in Table 4, Table 5 and Table 6). Highest here refers to the lowest true positive rate under consideration (not counting the light activity true positive rates in secondary optimization). In contrast, when optimized by PAP, the jerk (PAP) and acceleration (PAP) did not always demonstrate the predicted activity proportion most closely aligned with the gold standard (see overall rating for PMR rows in Table 4, Table 5 and Table 6). This indicates that the thresholds optimized by the TP approach may be the better choice.

For both optimization methods (TP or PAP), usually the acceleration is a marginally better derived quantity than jerk regardless of whether the sensor is worn on both legs or a single leg. Comparing the two optimization methods (TP or PAP) raises this question: For activity classification, is it more important to obtain a high number of true positives, or is it more important to correctly identify the overall predicted activity proportion? In other words, is it more important that the individual epochs match between the video and accelerometer data (TP) or that the overall summary of classifications into three different categories match (PAP)? The answer depends on the intended use of the method.

As expected, the thresholds derived from a single leg were approximately half that of the thresholds derived from sensors on both legs. For sensors on both legs, the TP method has an MTPR of 77.2–78.5%, while it is 67.8–73.1% for the left leg only and 77.1–78.5% for the right leg only. This performance is similar for both legs versus single leg. Similarly, for sensors on both legs, the PAP method has a PMR of 85.4–92.7%, while it is 87.6–91.5% for the left leg only and 87.6–89.3% for the right leg only. This performance is essentially the same. This indicates that using a sensor worn on a single leg to monitor physical activity levels is a reasonable approach. While using sensors on both legs can better represent the overall physical activity compared to using only one sensor on a single leg, the difference in performance of the algorithm is small, and it is less burdensome and requires less resources to use only a single sensor. Since the thresholds are different for the right and left sides, we propose using the right leg thresholds when using accelerometers on a single leg. The right leg thresholds perform slightly better (see above and Figure 2).

In cases where the user wants to simply describe the overall activity profile of an infant across a 24-h period, for example, we recommend the PAP method using acceleration (a typical use case). This would provide the most accurate information, for example, that an infant was sedentary for 60% of the time, in light of physical activity for 10% of the time, and in moderate-to-vigorous physical activity for 30% of the time. If the user wants a detailed description across time of when the infant was in different categories (describing each subsequent class of activity and its duration), then we recommend the TP method using jerk, as it was more accurate in identifying true positives.

It could be argued that, if the number of true positives is near perfect, the overall predicted activity proportion will also be near perfect. Thus, one should not have to choose between the two. However, obtaining near perfect true positives is not realistic, especially given the inherent limitations of this study described below.

For future work, we plan to use larger samples of infants to explore important research questions about infant physical activity. These include investigating differences in physical activity levels between TD versus AR infant groups, younger versus older infants, and periods with and without caregiver interaction.

We have developed a software tool based on the work presented in this paper. This enables other researchers to use their own accelerometer sensor data to compute physical activity intensity levels for infants. The software tool and usage instructions are available at https://github.com/Infant-Neuromotor-Control-Lab/pa-calc (accessed on 28 June 2024).

## 5. Limitations of This Study

This is a preliminary study with a small sample size. The thresholds were determined using a sample size of 10, and the observation duration was 2.71 to 8.45 min. To support generalizability, our diverse sample included pre-ambulatory infants with typical development and at risk across different ages who produced a variety of sedentary, light, and moderate-to-vigorous activities. However, the sample size limits the external validity of this study.

Another limitation is that this study used only sensors on the legs, so the sensors were not able to capture activity from the arms or head. This would result in a portion of sensor data (acceleration or jerk) that would actually be moderate-to-vigorous based on the gold standard (observational rating) but would appear to be light activity (see Figure 1d). This bias in the sensor data artificially increases the overlap between activity intensity level data and limits the maximum true positive rates achievable. This is an inherent limitation of using only two sensors on the ankles instead of using more sensors to capture movement of all limbs, the head, and the trunk. However, the algorithm performs with reasonable estimates, and it is less distracting for infants and less burdensome in terms of resources.

Although we are using observational rating of intensity of physical activity as our gold standard, it is a subjective rating by an observer. As is true with all sensor work using observation as a gold standard, the gold standard can be incorrect, creating cases where the objective sensor data are correct while the subjective observer data are incorrect. These erroneous instances can explain some of the differences in optimal performance of our two methods.

Finally, we do not consider here the potential effects of infants being moved around by the caregiver. In a previous study, we estimated that approximately 10% of what we counted as infant-produced leg movements using wearable sensors could actually be attributed to background motion produced by a caregiver [46].

## 6. Conclusions

In conclusion, an observational rating scale was developed to classify pre-ambulatory infant physical activity intensity levels into three groups: sedentary, light, and moderate-to-vigorous. Using video recordings from 10 infants, the observational rating scale was used to classify infant physical activity intensity. Using the observational ratings as the gold standard and using wearable accelerometer data from the same 10 infants, four sets of activity intensity thresholds were developed to classify infant physical activity intensity levels (for dual- and single-leg sensors). The most appropriate set of thresholds for a typical use case was found to be based on acceleration, developed using the PAP method, while wearing sensors on both legs (1.0 and 2.6 m/s). However, using a single sensor was deemed to be a reasonable approach when balancing considerations of accuracy vs. resource use. This is the first work to validate wearable accelerometer thresholds for activity intensity for pre-ambulatory infants.

## Figures and Tables

**Table 1:** Direct and indirect measures of physical activity intensity.

Measure	Type	Advantages	Limitations
Doubly labeled water (DLW)	direct	highly valid and reliable for estimating energy expenditure, unobtrusive, non-invasive	excessive cost, inability to capture duration of activity, participant burden, difficult logistics of multiple urine collections, multiple visits [4]
Maximal oxygen consumption (VO_2MAX_)	direct	highly valid, real-time or recorded data	expensive, difficult to carry, cumbersome to operate, potential safety issues for the very young [6]
Heart rate (HR) monitoring	direct	real-time or recorded data for long periods, unobtrusive, modest cost, relatively low participant burden	accuracy affected by emotional state, ambient temperature, fitness level, [4] muscle mass, [7] age, [4] can remain elevated after movement has stopped, i.e., lag [8]
Observational rating (video coding)	direct	flexible, provides contextual information, provides details on activities	high cost of time and energy, [9] potential for reactivity, [7] exists for children but not infants [3,5]
Wearable accelerometer	direct	real-time or recorded data for long periods, unobtrusive, small size, relatively modest cost, [4] relatively low participant burden	sensitivity of thresholds to age group and activity type, [10] potential for reactivity
Self-reported diaries	indirect	low cost, low participant burden	limited reliability [11,12]

Note. Ordered from most direct at the top to most indirect at the bottom.

**Table 2:** Details of infant participants and sensor data collected.

Participant Code	Group	Age (Days)	Body Length (cm)	Thigh + Shank Length (cm)	Aligned Session Length (min)
1	TD	31	53	18.5	5.96
2	TD	297	75.5	27.3	6.42
3	TD	65	61.5	23.6	8.45
4	TD	129	68.7	21.7	6.64
5	TD	47	59.8	25.7	4.69
6	TD	44	56	26	2.71
7	AR	137 *	59.5	27	5.76
8	AR	333 *	68.5	27.6	5.56
9	AR	202 *	67	32	6.11
10	AR	433 *	70	32	4.98

Note. Ages marked with a “*” indicate that they were adjusted for premature birth. TD group comprises infants who were typically developing. AR group comprises infants who were at risk for neurodevelopmental disabilities.

**Table 3:** Classification of epochs according to gold standard and by derived quantity threshold (accelerometer).

			**Accelerometer**		
			**Sedentary**	**Active**	
**Primary optimization step**	**Gold standard (observational rating)**	**Sedentary**	SED_SED_	SED_ACTIVE_	
		**Active**	ACTIVE_SED_	ACTIVE_ACTIVE_	
			**Accelerometer**		
			**Sedentary**	**Light**	**MV**
**Secondary optimization step**	**Gold standard (observational rating)**	**Sedentary**	SED_SED_	SED_LIGHT_	SED_MV_
		**Light**	LIGHT_SED_	LIGHT_LIGHT_	LIGHT_MV_
		**MV**	MV_SED_	MV_LIGHT_	MV_MV_
**Primary optimization step**	**Definitions:** SED_SED_: Sedentary, correctly predicted as sedentary SED_ACTIVE_: Sedentary, incorrectly predicted as active ACTIVE_SED_: Active, incorrectly predicted as sedentary ACTIVE_ACTIVE_: Active, correctly predicted as active				
**Secondary optimization step**	**Definitions:** SED_SED_: Sedentary, correctly predicted as sedentary SED_LIGHT_: Sedentary, incorrectly predicted as light SED_MV_: Sedentary, incorrectly predicted as MV LIGHT_SED_: Light, incorrectly predicted as sedentary LIGHT_LIGHT_: Light, correctly predicted as light LIGHT_MV_: Light, incorrectly predicted as MV MV_SED_: MV, incorrectly predicted as sedentary MV_LIGHT_: MV, incorrectly predicted as light MV_MV_: MV, correctly predicted as MV				

Note. These definitions were used in the evaluation of the derived quantity threshold during the primary optimization step (sedentary/active threshold) and secondary optimization step (light/MV threshold).

**Table 4:** Thresholds for the four approaches (combinations of derived quantities and optimization methods). Overall ratings based on validation dataset to demonstrate performance of thresholds (both legs).

**Threshold value**		**Threshold Name**	**Jerk, TP (m/s^2^)**	**Jerk, PAP (m/s^2^)**	**Acceleration, TP (m/s)**	**Acceleration, PAP (m/s)**
	**Primary optimization**	Sedentary/active	27.0	18.0	1.30	1.0
	**Secondary optimization**	Light/MV	41.0	56.0	1.80	2.6
**Overall rating, each threshold**		**Evaluation metric**	**Jerk, TP**	**Jerk, PAP**	**Acceleration, TP**	**Acceleration, PAP**
	**Primary optimization**	MTPR	78.4	71.5	78.5	76.4
		PMR	97.2	85.4	97.7	92.7
	**Secondary optimization**	MTPR	77.2	71.0	78.5	68.3
		PMR	83.1	85.4	78.0	92.7

Note: The derived quantities are area under jerk–time curve and area under acceleration–time curve. The optimum thresholds for each derived quantity were determined by either true positive (TP) rate or by predicted activity proportion (PAP) methods as described in the text. For overall rating, the primary optimization for sedentary/active and secondary optimization for sedentary/light/moderate-to-vigorous thresholds are shown. Minimum true positive rate (MTPR) is the overall rating for the TP method. PAP match rate (PMR) is the overall rating for the PAP method. Higher is better. Best methods in each row are highlighted.

**Table 5:** Thresholds for the four approaches (combinations of derived quantities and optimization methods). Overall ratings based on validation dataset to demonstrate performance of thresholds (left leg only).

**Threshold value**		**Threshold Name**	**Jerk, TP (m/s^2^)**	**Jerk, PAP (m/s^2^)**	**Acceleration, TP (m/s)**	**Acceleration, PAP (m/s)**
	**Primary optimization**	Sedentary/active	10.0	8.00	0.600	0.50
	**Secondary optimization**	Light/MV	16.0	27.0	0.800	1.30
**Overall rating, each threshold**		**Evaluation metric**	**Jerk, TP**	**Jerk, PAP**	**Acceleration, TP**	**Acceleration, PAP**
	**Primary optimization**	MTPR	70.1	65.3	67.8	70.8
		PMR	96.1	91.5	80.8	90.4
	**Secondary optimization**	MTPR	70.1	64.8	73.1	62.8
		PMR	84.2	87.6	74.1	90.4

Note: The derived quantities are area under jerk–time curve and area under acceleration–time curve. The optimum thresholds for each derived quantity were determined by either true positive (TP) rate or by predicted activity proportion (PAP) methods as described in the text. For overall rating, the primary optimization for sedentary/active and secondary optimization for sedentary/light/moderate-to-vigorous thresholds are shown. Minimum true positive rate (MTPR) is the overall rating for the TP method. PAP match rate (PMR) is the overall rating for the PAP method. Higher is better. Best methods in each row are highlighted.

**Table 6:** Thresholds for the four approaches (combinations of derived quantities and optimization methods). Overall ratings based on validation dataset to demonstrate performance of thresholds (right leg only).

**Threshold value**		**Threshold Name**	**Jerk, TP (m/s^2^)**	**Jerk, PAP (m/s^2^)**	**Acceleration, TP (m/s)**	**Acceleration, PAP (m/s)**
	**Primary optimization**	Sedentary/active	11.0	7.00	0.400	0.300
	**Secondary optimization**	Light/MV	17.0	24.0	0.800	1.00
**Overall rating, each threshold**		**Evaluation metric**	**Jerk, TP**	**Jerk, PAP**	**Acceleration, TP**	**Acceleration, PAP**
	**Primary optimization**	MTPR	78.5	70.8	77.1	71.5
		PMR	97.7	87.6	97.7	89.3
	**Secondary optimization**	MTPR	77.9	70.8	77.1	71.5
		PMR	81.4	87.6	78.0	89.3

Note: The derived quantities are area under jerk–time curve and area under acceleration–time curve. The optimum thresholds for each derived quantity were determined by either true positive (TP) rate or by predicted activity proportion (PAP) methods as described in the text. For overall rating, the primary optimization for sedentary/active and secondary optimization for sedentary/light/moderate-to-vigorous thresholds are shown. Minimum true positive rate (MTPR) is the overall rating for the TP method. PAP match rate (PMR) is the overall rating for the PAP method. Higher is better. Best methods in each row are highlighted.

**Table 7:** Comparison of measures of evaluation for all four approaches after each optimization step, based on the independent validation dataset (both legs).

			Gold Standard	Jerk, TP	Jerk, PAP	Acceleration, TP	Acceleration, PAP
**Primary optimization step**	**True positive rate (%)**	Sedentary	100	78.5	71.5	78.5	76.4
		Active	100	82.9	92.9	83.4	90.0
	**True negative rate (%)**	Sedentary	100	82.9	92.9	83.4	90.0
		Active	100	78.5	71.5	78.5	76.4
	**Predicted activity proportion (%)**	Sedentary Active	40.6	42.0	33.2	41.7	36.9
			59.4	58.0	66.8	58.3	63.1
**Secondary optimization step**	**True positive rate (%)**	Sedentary	100	78.5	71.5	78.5	76.4
		Light	100	25.8	54.5	18.2	51.5
		MV	100	77.2	71.0	80.0	68.3
	**True negative rate (%)**	Sedentary	100	82.9	92.9	83.4	90.0
		Light	100	93.4	83.0	94.8	84.4
		MV	100	72.4	76.7	69.5	78.1
	**Predicted activity proportion (%)**	Sedentary	40.6	42.0	33.2	41.7	36.9
		Light	18.6	10.1	23.9	7.61	22.3
		MV	40.8	47.9	42.8	50.7	40.8

Note: The metrics are true positive (TP) rate, specificity, and predicted activity proportion (PAP). Note that only TP and PAP were used for optimization.

**Table 8:** Comparison of measures of evaluation for all four approaches after each optimization step, based on the independent validation dataset (left leg only).

			Gold Standard	Jerk, TP	Jerk, PAP	Acceleration, TP	Acceleration, PAP
**Primary optimization step**	**True positive rate (%)**	Sedentary	100	70.1	65.3	76.4	70.8
		Active	100	76.3	83.4	67.8	72.0
	**True negative rate (%)**	Sedentary	100	76.3	83.4	67.8	72.0
		Active	100	70.1	65.3	76.4	70.8
	**Predicted activity proportion (%)**	Sedentary Active	40.6	42.5	36.3	50.1	45.4
			59.4	57.5	63.7	49.9	54.6
**Secondary optimization step**	**True positive rate (%)**	Sedentary	100	70.1	65.3	95.2	70.8
		Light	100	18.2	45.5	9.09	18.2
		MV	100	76.6	64.8	73.1	62.8
	**True negative rate (%)**	Sedentary	100	76.3	83.4	67.8	72.0
		Light	100	91.0	79.9	95.2	82.4
		MV	100	73.8	79.0	75.7	81.0
	**Predicted activity proportion (%)**	Sedentary	40.6	42.5	36.3	50.1	45.4
		Light	18.6	10.7	24.8	5.63	17.7
		MV	40.8	46.8	38.9	44.2	36.9

Note: The metrics are true positive (TP) rate, specificity, and predicted activity proportion (PAP). Note that only TP and PAP were used for optimization.

**Table 9:** Comparison of measures of evaluation for all four approaches after each optimization step, based on the independent validation dataset (right leg only).

			Gold Standard	Jerk, TP	Jerk, PAP	Acceleration, TP	Acceleration, PAP
**Primary optimization step**	**True positive rate (%)**	Sedentary	100	78.5	70.8	77.1	71.5
		Active	100	83.4	90.5	86.3	89.6
	**True negative rate (%)**	Sedentary	100	83.4	90.5	86.3	89.6
		Active	100	78.5	70.8	77.1	71.5
	**Predicted activity proportion (%)**	Sedentary Active	40.6	41.7	34.4	39.4	35.2
			59.4	58.3	65.6	60.6	64.8
**Secondary optimization step**	**True positive rate (%)**	Sedentary	100	78.5	70.8	77.1	71.5
		Light	100	13.6	37.9	10.6	31.8
		MV	100	77.9	71.7	80.0	73.8
	**True negative rate (%)**	Sedentary	100	83.4	90.5	86.3	89.6
		Light	100	91.7	82.0	91.7	83.7
		MV	100	71.0	75.2	67.6	73.8
	**Predicted activity proportion (%)**	Sedentary	40.6	41.7	34.4	39.4	35.2
		Light	18.6	9.30	21.7	8.73	19.2
		MV	40.8	49.0	43.9	51.8	45.6

Note: The metrics are true positive (TP) rate, specificity, and predicted activity proportion (PAP). Note that only TP and PAP were used for optimization.

## Data Availability

The data presented in this study are available on request from the corresponding author (B.A.S.) because a Material Transfer Agreement and/or Data Use Agreement with USC and/or CHLA may be required.
